# Contribution of Microbiota to Bioactivity Exerted by Bee Bread

**DOI:** 10.3390/ph17060761

**Published:** 2024-06-11

**Authors:** Nikos Asoutis Didaras, Ioanna Karaiskou, Marios Nikolaidis, Christina Siaperopoulou, Irini Georgi, Christina Tsadila, Katerina Karatasou, Grigoris D. Amoutzias, Dimitris Mossialos

**Affiliations:** 1 Microbial Biotechnology-Molecular Bacteriology-Virology Laboratory, Department of Biochemistry & Biotechnology, University of Thessaly, 41500 Larissa, Greece; didasout@yahoo.gr (N.A.D.); ikaraiskou@uth.gr (I.K.); siaperopoulou@uth.gr (C.S.); egeorgi@uth.gr (I.G.); tsadila@uth.gr (C.T.); 2 Bioinformatics Laboratory, Department of Biochemistry & Biotechnology, University of Thessaly, 41500 Larissa, Greece; marionik23@gmail.com (M.N.); amoutzias@uth.gr (G.D.A.); 3 Apicultural Centre of Larissa, Federation of Greek Beekeepers Associations, 41222 Larissa, Greece; omse@otenet.gr

**Keywords:** bee pollen, bee bread, microbiota, fermentation, bioactivity, antibacterial activity, enzyme production, pasteurization

## Abstract

Bee-collected pollen (BCP) and bee bread (BB) are honey bee products known for their beneficial biological properties. The main goal of this study was to investigate BB microbiota and its contribution to bioactivity exerted by BB. The microbiota of BB samples collected at different maturation stages was investigated via culture-independent (Next Generation Sequencing, NGS) and culture-dependent methods. Microbial communities dynamically fluctuate during BB maturation, ending in a stable microbial community structure in mature BB. Bee bread bacterial isolates were tested for phenotypes and genes implicated in the production and secretion of enzymes as well as antibacterial activity. Out of 309 bacterial isolates, 41 secreted hemicellulases, 13 cellulases, 39 amylases, 132 proteinases, 85 Coomassie brilliant blue G or R dye-degrading enzymes and 72 Malachite Green dye-degrading enzymes. Furthermore, out of 309 bacterial isolates, 42 exhibited antibacterial activity against *Staphylococcus aureus*, 34 against *Pseudomonas aeruginosa*, 47 against *Salmonella enterica* ser. Typhimurium and 43 against *Klebsiella pneumoniae*. Artificially fermented samples exerted higher antibacterial activity compared to fresh BCP, strongly indicating that BB microbiota contribute to BB antibacterial activity. Our findings suggest that BB microbiota is an underexplored source of novel antimicrobial agents and enzymes that could lead to new applications in medicine and the food industry.

## 1. Introduction

It has been suggested that honey bees evolved roughly 123 million years ago from carnivorous wasp species that replaced meat (as a food source) with pollen [1]. Bee-collected pollen (BCP), especially the fermented pollen designated bee bread (BB), is the main source of proteins, lipids, vitamins and minerals for honey bees. Furthermore, BB provides honey bees with valuable phytochemicals such as polyphenols [2,3].

The transition to a vegetarian diet was plausibly supported by the colonization of new microorganisms in the wasp gut, which facilitated pollen digestion [1]. A resilient outer layer consisting of a durable biopolymer called sporopollenin is present in pollen grains [4], which must be breached, to release essential nutrients bioavailable in the gut [5].

Highly nutritious pollen is not always abundant throughout the year [6]. Furthermore, pollen is a perishable food easily spoiled in the warm hive environment, due to its relatively high-water content and plethora of nutrients [7].

Bees have effectively dealt with all these issues by turning fresh pollen into BB, a fermented food that can be stored and preserved in the hive for a long time. Additionally, BB is considered more nutritious and bioactive compared to BCP and is considered a functional food [8,9,10]. 

BB consists of pollen derived from the plants growing in the vicinity of the hive, honey bee glandular secretions containing symbiotic microorganisms, added nectar and honey containing a heterogeneous community of microorganisms of plant and environmental origin [11,12,13,14]. Although BB is an important honey bee product, BB bioactivity and especially BB microbiota, is so far poorly investigated. Recent studies regarding BB bioactivity with a special focus on antimicrobial properties have been published [15,16,17]; however, BB microbiota and its putative contribution to bioactivity are still elusive. 

BB microbiota is important for honey bee nutrition and development [18,19]. It has been reported that BB microorganisms facilitate the enzymatic pre-digestion of pollen grains, thus increasing the digestibility and bioavailability of nutrients [20,21]. So far, the antimicrobial activity and biosynthetic potential of BB microbiota are largely unknown. Pelka et al., have shown that microorganisms isolated from BCP and BB produce antimicrobial compounds, as well as lipolytic, proteolytic, cellulolytic and esterolytic enzymes [22]. 

Furthermore, it has been demonstrated that BB exerted significantly higher antimicrobial activity than pollen [17]. Similarly, the antibacterial activity exerted by BB in water suspensions against clinically important pathogens was remarkable [16]. The application of chemical solvents (ethanol, methanol, ether), or the implementation of other processing techniques such as sonication, leads to breached pollen grains, thus increasing the availability of known antimicrobial phytochemicals such as polyphenols. The finding that virtually unprocessed BB demonstrated similarly high or even higher antimicrobial properties led us to hypothesize the presence of other than polyphenol antimicrobial agents, presumably produced by BB microbiota [16]. In a similar way, it has been recently reported that BB water suspensions exerted significant antiviral activity against Enterovirus D68 and Influenza A Virus (IVA) H1N1 [23,24].

BB microbiota consists of both plant and bee-derived microorganisms, but it seems that those of bee origin are relatively more abundant. It has been reported that changes in BCP microbiological and biochemical composition start as soon as pollen is collected [20]. Given that the nine bacterial phylotypes, which constitute the core gut microbiome of adult worker bees, are poorly represented in BB [18,25], it suggests that mainly microorganisms derived from honey bee stomach, including a variety of lactic acid bacteria, are present in BB microbiota [26,27].

Forager bees inoculate flower pollen with lactic acid bacteria (LABs) that are present in their honey stomach, an organ used to contain foraged nectar or water to be brought to the nest and for trophallaxis, the sharing of nectar among nest mates, which is part of honey production [11,28]. Indeed, forager bees regurgitate nectar and add it to collected pollen grains along with their secretions to form the pollen pellets they carry back to the hive. The pollen pellets end up in the comb cells where they are further processed by middle-aged bees by adding honey and glandular secretions. Finally, bees push the pollen pellets with their heads to pack them tightly into the cell, where BCP undergoes solid-state fermentation [29]. 

It has been suggested that BCP microorganisms are present inside the comb cell under aerobic, anaerobic and microaerophilic conditions [30], similar to microorganisms isolated from BB [31,32,33,34,35] that produce organic acids through both anaerobic and aerobic fermentation [36,37]. 

Researchers investigated the diversity of LABs previously isolated from the honey stomach of bees, in BCP samples, in two-week- and two-month-old BB samples. They identified twelve bacterial species belonging to *Lactobacillus* and *Bifidobacterium* genera. The majority of them remained viable in BCP and in two-week-old BB but not in older BB samples [11,38]. LABs might be considered as a starter culture of BCP fermentation [11,29] and they are known to produce antimicrobial compounds, thus contributing towards BB preservation in the hive [11,39]. Yeasts are also present in BB [29,31,32] and could similarly be implicated in BCP fermentation. 

Recently, Dranca et al. [37] reported that lactic acid was not detected in BB but instead gluconic acid was the most abundant organic acid. They reported that mature BB contained 97.9 g/kg organic acids, with gluconic acid amounting to 79.2 g/kg, followed by acetic acid (10.4 g/kg) and formic acid (6.75 g/kg). Propionic acid (1.30 g/kg) and butyric acid (0.33 g/kg) were also detected [37]. Gluconic acid is produced from glucose either enzymatically by glucose oxidase, usually produced by fungi, or by glucose dehydrogenase produced by bacteria such as *Gluconobacter*, through aerobic fermentation [40]. 

Overall, BB contains microbial communities that flourish or perish during BB maturation. Gilliam [20] was the first to suggest that, during the transformation of BCP to BB, microbial succession takes place. Recent research has demonstrated that the diversity and community structure of BB microbiota indeed fluctuates during maturation [29,32,41,42].

The main goal of this study is to investigate how BB microbiota contributes towards BB bioactivity and identify the most relatively abundant microbial groups using both culture-independent (Next Generation Sequencing, NGS) and culture-dependent methods, starting from early fermentation to maturation. Moreover, it has been tested whether specific enzymes, such as amylases, proteinases, xylanases, cellulases and laccases, that presumably facilitate the digestion of pollen grains, could be produced by culturable microorganisms isolated from BB. Finally, the antimicrobial activity of BB isolates was assessed against important human pathogens, to elucidate the putative contribution of microbiota towards the high antimicrobial activity exerted by BB. Regarding this aspect, we fermented double pasteurized fresh BCP using BB isolates as a starter culture and then we tested the antimicrobial activity exerted by the fermented BCP. 

## 2. Results

### 2.1. Metataxonomic Microbiota Analysis during BB Maturation

Metataxonomic analysis implementing Next Generation Sequencing (NGS) was performed to elucidate the microbiota dynamics during BB maturation. The basic statistics of each sample are shown in Supplementary Table S1. Rarefaction curves indicated that sequencing depth was sufficiently high regarding all samples (Supplementary Figures S1 and S2).

**Figure 1:**
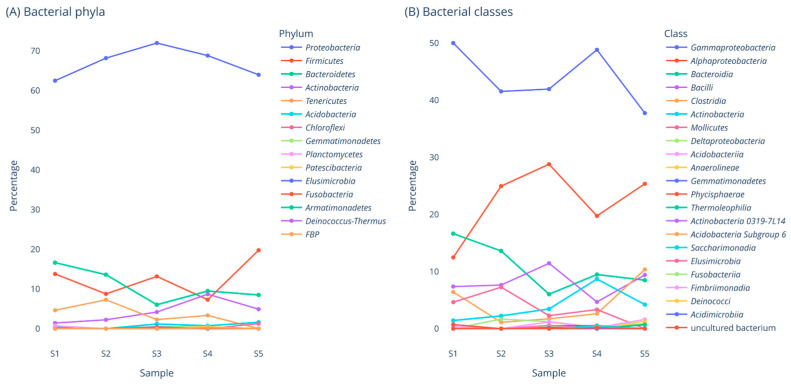
Bacterial phyla and classes detected via NGS in samples S1 to S5.

**Figure 2:**
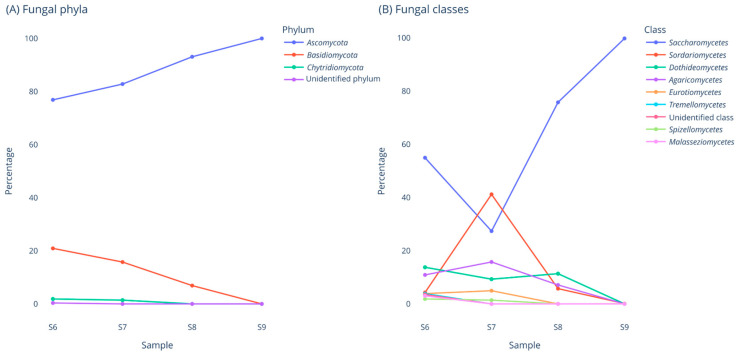
Fungal phyla and classes detected via NGS in samples S6 to S9.

At the beginning of BB maturation, bacterial diversity fluctuated as depicted by Chao 1 index values: 46 (1-day-old BCP), 35 (11-day-old BB), 82 (27-day-old BB) and 42 (41-day-old BB) (see Supplementary Table S2). Regarding diversity and evenness, high fluctuation of Shannon index values was not observed, with a notable exception of samples S1 and S2 (Supplementary Table S2). Shannon index values were the following during maturation: 5.121 (1-day-old BCP), 4.778 (1-day-old BB), 5.714 (11-day-old BB), 5.603 (27-day-old BB) and 5.146 (41-day-old BB). Fungal diversity reduced gradually from day 1 to day 27, displaying Chao 1 index values of 26 (1-day-old BCP), dropping to 8 (27-day-old BB) and Shannon index values from 3.380 (1-day-old BCP) to 0.586 (27-day-old BB) (see Supplementary Table S3). These findings clearly indicate that during BB maturation, microbiota diversity dynamically fluctuates, ending up in a rather stable microbial community structure. Beta-diversity indices (Supplementary Tables S6–S13) showed no significant differences between samples. Figure 1 and Figure 2 depict bacterial and fungal phyla as well as classes present in samples S1 to S9, while Supplementary Tables S4 and S5 summarize the most dominant (relative abundance >3%) families and genera of bacteria and fungi, respectively. Beta-diversity metric tables for bacteria are shown in Supplementary Tables S6–S9 and, for fungi, in Supplementary Tables S10–S13. Proteobacteria is the dominant phylum in BCP (S1), representing more than 60% of total operating taxonomic units (OTUs). Proteobacteria relative abundance increased rapidly within 24 h (S2), reaching almost 70% and gradually declined from day 11 to day 41, reaching approximately the initial relative abundance (S3–S5). Gammaproteobacteria were dominant (50% of total OTUs in S1) but dropped below 40% in S5. *Pseudomonas* genus abundance was significantly variable during BB maturation (S1: 4.62%, S2: 1.12%, S3: 6.1%, S4: 14.14%, S5 BB: 4.68%). The sharp decline from 4.62% to 1.12% within the first 24 h might be attributed to initial BB fermentation and honey inoculation. After that time, there is a progressive increase (days 11 to 27) and finally, a significant decrease from day 27 (S4) to day 41 (S5), possibly because BB was fully matured. From day 12 till day 41, the sampling frame was isolated within the hive so the honey bees could not add BCP or consume ΒΒ.

Alphaproteobacteria constitute a significant class, amounting up to 30% in S3 but dropped to 25% in S5. *Sphingomonas* was a bacterial group that its abundance increased from 6.24% (S1) to 13.34% (S5).

Firmicutes relative abundance increased to 20% in S5. Bacilli and Clostridia are both dominant groups representing roughly 20%. Clostridia declined rapidly within the first 24 h and then progressively increased till day 41. On the contrary, *Lactobacillus* genus increased rapidly within the first 24 h, indicating the contribution of Lactobacilli in the fermentation process, especially at the very beginning. The fermentation process is crucial for BCP transformation to BB. Of note, *Lactobacillus* spp. are still present in 41-day-old BB, indicating their adaptability in this environment. Bacteroidetes is also an abundant phylum whose abundance gradually decreased.

In this study, NGS data demonstrated that *Sphingomonas, Pseudomonas, Acinetobacter* and *Lactobacillus* are important bacterial genera implicated in BB maturation as their relative abundance fluctuated at different maturation stages (Supplementary Table S4). These genera were present in S5 (41-day-old BB), each one representing more than 4% of total OTUs (Supplementary Table S4). *Enterobacter* spp. increased from 4.62% in one-day-old BCP (sample S1) to 7.08% in 1-day-old BB (sample S2). However, they were not detected in 11-day-old BB (sample S3). Furthermore, an unidentified genus of Enterobacteriaceae was detected in BB, ranging from 1.69% to 9.24% of OTUs, and remained present in 41-day-old BB (5.36%). *Massilia*, a bacterial genus detected in different environments but mostly related to plants, was the most abundant genus in the early BB fermentation stages (13.06% in 1-day-old BCP), dropping to 2.93% in 41-day-old BB (sample S5). 

Regarding fungi, Ascomycota was the most abundant phylum, representing almost 100% of related OTUs in 27-day-old BB (Supplementary Table S5). The most dominant Ascomycota classes were *Saccharomycetes* and *Sordariomycetes*. Agaricomycetes relative abundance was almost 18% in sample S7 (1-day-old BB) and dropped to zero in sample S9 (27-day-old BB). Similarly, Dothideomycetes relative abundance was close to 15% in samples S6, S7 and S8, and dropped to zero in sample S9. *Zygosaccharomyces* was the most abundant genus, amounting to 99.58% of related OTUs in 27-day-old BB (S9 sample). Basidiomycota was the second most abundant fungal phylum, though its relative abundance decreased gradually from 20% in sample S6 (1-day-old BCP) to 0% in sample S9 (27-day-old BB).

### 2.2. Antimicrobial Activity and Biosynthetic Potential of Isolated BB Bacteria

Bacterial isolates (isolated from S10 and S11) were screened for antibacterial activity against four pathogens (*S. aureus*, *P. aeruginosa*, *S. enterica* ser. Typhimurium and *K. pneumoniae*), as well as for enzyme production, i.e., secretion of cellulases, hemicellulases, amylases, proteinases and oxidases, among them, laccases that are capable to degrade dyes. Isolates that exerted antibacterial activity at least against two pathogens and simultaneously produced diverse enzymes were selected for identification through 16S rRNA gene sequencing. Furthermore, we identified seven isolates that exerted antibacterial activity against one pathogen and three isolates that did not exert any antibacterial activity but still were able to produce various enzymes. The identified strains, the antibacterial activity and biosynthetic potential are presented in Table 1 and Table 2. Overall, eleven strains were identified as *Apilactobacillus kunkeei*, one strain as *Fructobacillus fructosus*, one strain as *Pseudomonas αeruginosa*, one strain as *Staphylococcus hominis*, five strains as *Bacillus thuringiensis*, three strains as *Bacillus cereus*, three strains as *Bacillus siamensis*, two strains as *Bacillus licheniformis*, three strains as *Bacillus subtilis*, five strains as *Bacillus safensis*, one strain as *Bacillus halotolerans*, one strain as *Bacillus pumilus*, one strain as *Bacillus aerophilus* and 10 strains were identified at genus level as *Bacillus* spp.

### 2.3. Antibacterial Activity Exerted by BB Isolates

Out of 309 bacterial isolates, 42 exhibited antibacterial activity against *S. aureus* MRSA strain 1552, 34 against carbapenem-resistant *P. aeruginosa* strain 1773, 47 against *S. enterica* ser. Typhimurium and 43 against *K. pneumoniae*. 

*Apilactobacillus* and *Fructobacillus* strains exerted higher antibacterial activity against Gram-negative pathogens (inhibition zone ranged from 1.8 cm to 3.5 cm, see Table 1). However, these strains exerted less antibacterial activity against Gram-positive *S. aureus*. *Bacillus* isolates demonstrated antibacterial activity against mainly *S. aureus*, though some exerted antibacterial activity against Gram-negative pathogens (Table 2). 

### 2.4. Biosynthetic Potential of BB Isolated Bacteria

Out of 309 bacterial isolates, 41 secreted hemicellulases, 13 cellulases, 39 amylases, 132 proteinases, 85 Coomassie brilliant blue G or R dye-degrading enzymes and 72 Malachite Green dye-degrading enzymes. All identified strains were producing more than one of the above enzymes (Table 2). 

Corresponding genes were partially amplified through PCR to investigate the bacterial gene content associated with laccase and hemicellulase production. The endoxylanase gene (336 bp fragment) was amplified from isolates, demonstrating a strong positive xylanase phenotype. Out of 35 tested isolates, 20 of them were positive, whereas 15 were negative, thus indicating the presence of various xylanolytic enzymes was probably exhibiting different modes of activity [43]. Furthermore, amplicons of ON911245 (*Bacillus* sp.), ON911255 (*Bacillus* sp.), ON911280 (*B. safensis*) and ON911281 (*B. safensis*) isolates were sequenced and indeed confirmed as endoxylanase gene fragments (Supplementary Table S14). 

The lacasse gene (142 bp fragment) was amplified via PCR from isolates, demonstrating a strong dye degradation phenotype. Out of 55 tested isolates, 22 were positive, whereas 33 were negative. These findings indicate the presence of other dye-degrading enzymes such as lignin peroxidase, manganese peroxidase, versatile peroxidase and dye peroxidase [44]. Laccase activity has been detected on the guaiacol substrate. Positive colonies turned brown as expected and the surrounding substrate was turned light brown. Therefore, we assume that laccase-positive isolates may produce membrane-bound laccases. PCR amplicons of strains ON911251 (*B. subtilis*), ON911253 (*B. cereus*) and ON911267 (*B. thuringiensis*) were sequenced, thus confirming the presence of laccase gene in positive strains (Supplementary Table S14).

Cellulase and hemicellulase phenotypes were also directly detected in the protein fractions of BB, which further supports the hypothesis that BB microbiome facilitates the predigestion of pollen grains.

### 2.5. Antibacterial Activity Exerted by Double Pasteurized and Artificially Fermented BCP

The unpasteurized BCP (AF1) MIC has been determined at 12.5% *w*/*v* regarding pathogens *S. aureus* and *S. enterica* ser. Typhimurium, whereas MBC values were >12.5% *w*/*v* (Table 3). The MIC value of AF46D (non-inoculated, double pasteurized AF1) against *S. aureus* did not alter, thus implying that the antibacterial compounds inhibiting *S. aureus* growth were heat resistant. In contrast, AF46D MIC value was >12.5% *w*/*v* against *S. enterica* sub. Typhimurium, implying that the antimicrobial compounds inhibiting this pathogen were heat-labile. We assumed that due to fermentation, antimicrobial compounds contributing to exerted antibacterial activity might be produced. Τhis hypothesis is further supported by the MIC and MBC values of AF42D, AF43D and AF 44D against *S. enterica* ser. Typhimurium. AF42D MIC value (6.25% *w*/*v*) is lower compared to AF46D and AF1 (MIC 12.5% *w*/*v*), thus demonstrating higher antibacterial activity. *A. kunkeei* strain NADBB37 was used as starter culture in AF42D. A different *A. kunkeei* strain (NADBB7) was used as a starter culture in AF43D. AF43D demonstrated the same MIC value compared to AF42D. However, AF43D (in contrast to AF42D) demonstrated bactericidal activity against *S. enterica* ser. Typhimurium (MBC 12.5% *w*/*v*). It is plausible that *A*. *kunkeei* NADBB7 produces different or at higher concentration antimicrobial compounds against *S. enterica* sub. Typhimurium compared to *A. kunkeei* NADBB37. A starter culture containing a mixture of two bacterial strains and a yeast (*A. kunkeei* NADBB37, *Fructobacillus fructosus* and *Zygosaccharomyces siamensis*) was implemented in fermentation of AF44D. It has been shown that AF44D did not exert bactericidal activity against *S. enterica* sub. Typhimurium (MBC > 12.5% *w*/*v*) but only bacteriostatic activity (MIC 12.5% *w*/*v*).

A starter culture consisted of *A*. *kunkeei* NADBB37 and prolonged fermentation time was applied to AF5U (29 days at 33 °C). AF5U MIC and MBC values were the lowest in this study against both tested pathogens (Table 3).

Our data have shown that the BCP lost its antibacterial activity against *Salmonella enterica* ser. Typhimurium after the double pasteurization process. Following fermentation, the artificially fermented BCP re-gained antibacterial activity against the tested pathogens, indicating that BB microbiota might contribute to antimicrobial activity either by antibacterial compound production or by releasing antibacterial compounds during the fermentation of BCP grains. Our data clearly indicate that the type of starter culture as well as fermentation conditions both affect the antibacterial activity of artificially fermented BCP (Table 3).

## 3. Discussion

Our findings indicate that during BB maturation, microbiota diversity dynamically fluctuates, ending up in a rather stable microbial community structure. Changes affecting microbial growth (production of metabolites, oxygen concentration, acidity, nutrients and water availability) might occur during BB maturation, thus forming a highly variable environment. Therefore, we assume that the observed variability regarding the diversity and abundance of specific microbial groups is due to antagonism or adaptation taking place during BB maturation.

Our data are in accordance with the study conducted by Ghosh et al. [30]. They compared BCP and BB bacterial diversity by implementing NGS (Illumina MiSeq) sequencing. Samples were collected from *Apis mellifera ligustica* colonies in Andong, Korea. The dominant phyla were Proteobacteria, followed by Firmicutes in BB [30]. However, Firmicutes were the most predominant phyla in BCP. Detected Firmicutes orders were Bacillales, Lactobacillales and Clostridiales. Lactobacillales were present only in BCP. 

Similarly, Donkersley et al. [33] implemented NGS (Illumina MiSeq) sequencing to investigate the microbial diversity of North West England BB samples of unknown age collected from apiaries. They reported that Proteobacteria was the most dominant phylum, followed by Bacteroidetes and Firmicutes. *Pseudomonas* (32.4%), *Arsenophonus* (13.0%), *Lactobacillus* (8.2%), *Erwinia* (7.7%) and *Acinetobacter* (5.2%) were the most common genera. *Massilia* was identified in BB in relatively low abundance along with *Sphingomonas* [33].

Regarding fungi, our data showed that Ascomycota was the most abundant phylum, representing almost 100% of related OTUs in 27-day-old BB (Supplementary Table S5). The most dominant Ascomycota classes were *Saccharomycetes* and *Sordariomycetes*. *Zygosaccharomyces* was the most abundant genus, amounting to 99.58% of related OTUs in 27-day-old BB (S9 sample). Basidiomycota was the second most abundant fungal phylum, though its relative abundance decreased gradually from 20% in sample S6 (1-day-old BCP) to 0% in sample S9 (27-day-old BB). Our findings are in accordance with data reported in previous studies. Dimov et al. applied NGS-based metataxonomics (Illumina HiSec) to investigate the mycobiome of 2- to 4-day-old BB, collected from four locations in Bulgaria. All dominant genera belonged to the Ascomycota phylum (>99%) [45]. Sinpoo et al. combined PCR, DGGE and culture-dependent methods to identify fungal communities in BCP and 1-, 2-, 3-, 4- and 6-week-old BB in Thailand, whereas the bees foraged pollen mainly from *Mimosa pudica* L. Fungal populations and diversity decreased with storage. The dominant fungal species in BB were yeasts of the genus *Zygosaccharomyces* and filamentous fungi of *Cladosporium* and *Aspergillus* genera [41]. Similarly, Detry et al. isolated yeasts from 7-day-old and 20-day-old BB samples collected from apiaries in Belgium. Isolates were identified via PCR and analysis showed that most of the 252 identified isolates belonged to the genera *Starmerella*, *Metschnikowia* and *Zygosaccharomyces*. *Starmerella* (*Candida*) *apis* was the most dominant species in fresh BB, while *Zygosaccharomyces* spp. were more abundant in mature BB [42].

BB bacterial isolates were screened for antibacterial activity as well as for the production of diverse enzymes. Isolated bacteria demonstrated positive phenotypes regarding the secretion of cellulases, amylases and proteinases in accordance with a previous study [22]. Moreover, this is the first study reporting the secretion of hemicellulases, Coomassie Brilliant Blue G or R dye-degrading enzymes and Malachite Green dye-degrading enzymes by BB bacteria. The production of lignin-degrading enzymes (laccases or oxidases) by BB bacteria might promote exine (the outer layer of pollen grains) degradation. Moreover, the production of cellulases is essential for intine (the inner layer of pollen grains) degradation, which mainly consists of cellulose [46]. The ability of BB bacteria to breach pollen grain walls facilitates the release of nutrients and bioactive compounds contained in pollen grains. Recently, Damulienė et al. reported that enzymatic hydrolysis of BCP increased total phenolic content by 1.1 to 2.5 times, total flavonoid content by 1.1 to 3.0 times, radical scavenging activity by 1.1–3.5 times and antibacterial activity by 1.1 to 3.3 times [47]. Iorizzo et al. investigated LAB communities in BB as well as honey stomach and midgut of *Apis mellifera ligustica* in Italy. Three RAPD_isolates (Lpla.I, Lpla.II and Lpla.VI) present exclusively in BB, produced esterase, esterase lipase, α-glucosidase and β-glucosidase. RAPD_biotypes Lpla.I and Lpla.VI were also able to utilize raffinose, while the RAPD_isolate II metabolized melezitose [48]. Rokop et al. implemented culture -based methodology and molecular taxonomy to identify LAB communities present in different hive milieu. Both *Fructobacillus* spp. and *Lactobacillaceae* were isolated from brood cells, BB and nectar, and co-culture assays showed that these bacteria promote the growth of honey bee core bacterial species. Metabolic characterization of *Fructobacillus* spp. showed that these species could utilize simple sugars as well as the complex carbohydrate lignin [49]. Di Cagno et al. demonstrated that spontaneous fermentation of BB, after BCP inoculation with selected *L. kunkeei* strains and the yeast *Hanseniaspora uvarum* AN8Y27B, increased digestibility as well as bioavailability of nutrients and bioactive compounds naturally occurring in BCP. Artificially fermented BCP contained higher concentrations of peptides, free amino acids and free phytochemicals [29]. 

Investigation on biosynthetic potential (secondary metabolites and enzymes) of bacteria isolated from BB is underexplored. We have demonstrated that bee bread bacteria produce enzymes that could degrade pollen grains through the maturation process, thus releasing nutrients and bioactive compounds, leading to increased bioavailability. BB quality, nutritional value and potential health benefits for bees and humans depend indeed on the BB maturation process. 

Further research could lead to biotechnological applications and a better understanding of BCP conversion to BB. Future research could explore the bioactive compounds produced by BB microbiota, and investigate the metabolic profile and the impact of environmental factors on BB composition in relationship with bee health and performance.

*Apilactobacillus* and *Fructobacillus* strains exerted high antibacterial activity against *Gram*-negative pathogens but exhibited lower antibacterial activity against *S. aureus*. On the other hand, *Bacillus* isolates demonstrated antibacterial activity against mainly *S. aureus*, though some exerted antibacterial activity against Gram-negative pathogens. It is plausible that BB antibacterial activity is the outcome of plant metabolites, such as polyphenols and antimicrobial compounds produced by BB microbiota [15]. LABs are known to produce antimicrobial compounds such as lactic acid, acetic acid, hydrogen peroxide, reuterin, diacetyl, reutericyclin, acetoin and more complex molecules like bacteriocins and antifungal peptides [39,50,51]. In a previous study, diverse Bacilli isolates were reported to exert antibacterial activity against *S. aureus* [22]. However, some of the isolates tested in this study exerted antibacterial activity against Gram-negative pathogens too. To our best knowledge, this is the first study demonstrating the antibacterial activity of *Apilactobacillus* and *Fructobacillus* strains isolated from BB. 

In order to further investigate the contribution of BB microbiome to antibacterial activity, autochthonous microbial strains, isolated from BB samples, were implemented as a start culture in fermentation of double pasteurized BCP. It has been reported by several authors that BCP fermentation ameliorates BCP biological properties in terms of nutrient composition, bioactive components such as polyphenols and even flavor [7,8]. Potential health benefits include increased bioavailability of nutrients, prebiotic and probiotic potential and the possible degradation of allergenic compounds [29,52]. Kaškonienė et al. studied the impact of spontaneous and artificial solid-state lactic acid fermentation of natural and pasteurized BCP on its antibacterial, antifungal and antioxidant activities. *Lactococcus lactis* and *Lactobacillus rhamnosus* were used as starter cultures. It has been demonstrated that following BCP fermentation, total phenolic content, total flavonoid content and radical scavenging activity increased as well as antibacterial and antifungal activity against *Micrococcus luteus*, *Staphylococcus aureus*, *Escherichia coli* and *Penicillium roqueforti* respectively. Pasteurized BCP showed significantly lower antimicrobial and antioxidant activities after fermentation than unprocessed fermented BCP, indicating that BCP natural microbiota further contributes to antimicrobial and antioxidant properties exerted by artificially fermented BCP [21]. Urcan et al. showed that it is feasible to enhance the antioxidant and antimicrobial activity of BCP through solid-state fermentation [53]. Similarly, a recent study reported that microbiota enhanced BB antiviral activity against Influenza A virus, as shown by the higher antiviral potential of artificially fermented BCP compared to fresh unfermented BCP [23]. 

We implemented a double pasteurization protocol to eliminate most of the natural microbiota, thus demonstrating unequivocally the indigenous microbial contribution (starter culture) to antimicrobial activity exerted by BB. Our artificially fermented samples exhibited higher antibacterial activity compared to double pasteurized BCP as well as fresh BCP (unpasteurized) against *S. aureus* and *S.* Typhimurium, indicating that indigenous BB microbiota contributes towards BB bioactivity. To the best of our knowledge, this is the first study using double pasteurized BCP in solid-state fermentation. 

## 4. Materials and Methods

### 4.1. Sampling 

Out of 11 samples (Table 4), 10 were harvested in situ from bee colonies located in Mt Pelion, an area known for high plant diversity, attracting many beekeepers. Sample 11 was collected from a hive frame containing spring BB, provided by a beekeeper from Rethymno, Crete. 

Sampling was performed after the placement of empty, marked honeycombs in the beehives. The surface of each honeycomb was divided into five segments. Sampling was performed (from each segment) during a different time point. Briefly, 1-day-old BB (S2 and S7) was collected on day one from one out of the five segments, 11-day-old BB (S3 and S8) was collected from another segment, and so on for BB 27-day-old (S4 and S9) and 41-day-old (S5 and S10). The honeycombs were filled with BCP in a few days, so it was plausible to figure out the age of the BB. BCP was collected using entrance traps after 24 h (BCP 1 day old, samples S1 and S10). Samples S1 and S6 originated by dividing the same initial sample, the same applies to samples S2 and S7, samples S3 and S8 and samples S4 and S9, respectively. 

Samples were collected aseptically in order to elucidate the diversity and relative abundance of microbial communities at different stages of BB maturation. From day 12 the BB comb used for sampling was isolated inside the hive environment (using a frame containing a metal net without touching the comb), so that the bees could not add BCP to stored BB or consume it (samples S4, S5, S9). Bacteria were isolated and cultured from samples S5 and S11. Although melissopalynological analysis was not performed, beekeepers provided information regarding the botanical origin of collected pollen. Regarding samples S1–S9, the most abundant plant families were Asteraceae, Lamiaceae, Cistaceae, Brassicaceae, Ranunculaceae and Papaveraceae, whereas regarding S11 sample, the most abundant plant families were Lamiaceae, Cistaceae, Brassicaceae, Ranunculaceae and Papaveraceae. Similarly, the AF1 sample, according to the beekeeper who provided it, contained mostly pollen derived from Brassicaceae, Ranunculaceae, Papaveraceae and Cistaceae.

### 4.2. Metataxonomic Analysis during BB Maturation

Total DNA was extracted from 0.2 g of S1–S10 samples using a Macherey Nagel NucleoSpin Food kit (Hoerdt, France) according to the manufacturer’s instructions. Extracted DNA was used as a template to amplify the V3–V4 region of the 16S rRNA gene, using the primers 338F (5′-ACTCCTACGGGAGGCAGCA-3′) and 806R (5′-GGACTACHVGGGTWTCTAAT-3′). Similarly, the ITS1 region of fungi was amplified using the ITS1-1F-F (5′-CTTGGTCATTTAGAGGAAGTAA-3′) and ITS1-1F-R (5′-GCTGCGTTCTTCATCGATGC-3′) set of primers. Amplicon quality was assessed via agarose gel electrophoresis and Qubit assay. PCR products were used to construct an amplicon library. Subsequently, purified amplicons were paired-end sequenced on an Illumina MiSeq PE250 platform (Macrogen, Republic of Korea). Raw sequence reads were deposited into the NCBI Sequence Read Archive (SRA) database (Bioproject: PRJNA846146, BioSample Accession Numbers: SAMN28869225-SAMN28869233).

### 4.3. Bioinformatics Analysis

Bioinformatics analysis of raw sequencing data was performed with the QIIME2 platform [54]. Denoising, de-replication and chimera filtering were performed with the DADA2 plugin [55], whereas the bacterial sequences were trimmed (after manual inspection of the FASTQ scores) at 290 and 220 nucleotides long for forward and reverse reads, respectively. The fungal forward and reverse reads were trimmed at 240 and 180 nucleotides long, respectively. The chimera filtering was based on the default consensus method. Next mitochondrial, chloroplastic and non-assigned sequences were removed. Diversity analysis was performed with a sampling-depth cutoff of 530 for 16S and 890 for ITS. OTU clustering was based on a 97% sequence identity cutoff. Alpha- and beta-diversity metrics were calculated using the qiime diversity module. Faith, Shannon, observed OTUs, Chao1 and Evenness Alpha-diversity core metrics were calculated. Unweighted unifrac distance matrix, weighted unifrac distance matrix, Jaccard distance matrix, Bray–Curtis distance matrix and Beta-diversity metrics were also calculated. Alpha rarefaction was estimated with a maximum depth of 530 for 16S and 890 for ITS.

Taxonomic classification was based on the 16S Silva database version 132 (SILVA_132_97_16S.fna) [56] and UNITE ITS database version 8 (ver8_97_02.02.2019) [57] with the Vsearch algorithm, where the taxonomy cutoff was based on 97% nucleotide identity. In order to identify the core microbiota a filter was applied where the abundance of each genus should be at least 1% of the assigned sequences.

### 4.4. Isolation of BB Culturable Bacteria 

Bacteria were isolated by spreading 100 μL of BB sterile saline dilutions (10^−1^, 10^−2^ and 10^−3^) on six agar plates of different culture media. Three out of six agar plates were incubated at 37 °C and the rest at 30 °C at least for 5 days. Culture media to isolate diverse bacterial groups were the following: Plate Count Agar (PCA) (Neogen, Heywood, UK), *Bacillus cereus* medium (LAB M, Bury, UK), containing 1.5% bacteriological agar and polymyxin B (LAB M, Bury, UK) were incubated aerobically. Man Rogosa and Sharpe (MRS) agar plates (NEOGEN, Heywood, UK) were incubated anaerobically in an AnaeroJar AG25 with the AnaeroGen Atmosphere Generation system (Oxoid, Basingstoke, UK) at 30 °C for approximately 5 days. In the case where several morphologically identical colonies were developed on a culture medium, at least three of them were picked up. Pure cultures were stored as glycerol stocks in Nutrient Broth (Biolab, Budapest, Hungary) supplemented with 0.5% *w*/*v* Yeast Extract (Neogen, Heywood, UK), 0.5% *w*/*v* glucose (SERVA, Heidelberg, Germany) and 20% *w*/*v* glycerol (SIGMA-ALDRICH, St. Louis, MO, USA) at −80 °C until further analysis.

### 4.5. Assessment of Antibacterial Activity Exerted by BB Bacterial Isolates

The method of cross streaking was performed to assess the antibacterial properties of isolated BB bacteria against human pathogens. BB isolates were inoculated on PCA plates in a line. After incubation long enough to allow growth (at least 5 mm wide) of the tested isolates, the pathogens were streaked perpendicularly, followed by incubation at 37 °C for 24 h. Broth cultures of pathogens, which were set up at OD 600 nm: 0.1, were further diluted 1/10 in Mueller–Hinton (NEOGEN, Heywood, UK) and implemented in cross streaking assay. The growth inhibition of pathogens in terms of distance from BB isolates was measured and recorded. The following pathogens were tested: methicillin-resistant *Staphylococcus aureus* 1552, carbapenem-resistant *Pseudomonas aeruginosa* 1773, *Salmonella enterica* serov. Typhimurium and *Klebsiella pneumoniae* [58]. 

### 4.6. BCP Double Pasteurization-Solid State BCP Fermentation

Multifloral bee-collected pollen (BCP) was collected in Thessaly, Greece in spring 2022, (sample AF1). 

The double pasteurization process was performed as follows: AF1 BCP was pasteurized at 65 °C for 40 min in a water bath. In total, 11.5 gr of AF1 sample was mixed with 1 mL NB3 [NB3 is composed of Nutrient Broth containing 0.5% *w*/*v* yeast extract (Neogen, Heywood, UK) and 0.5% *w*/*v* glucose (SERVA, Heidelberg, Germany)] in a sterile 50 mL tube. After pasteurization, the tube was incubated at 33 °C for 48 h allowing bacteria that survived to grow. Then, a second pasteurization (65 °C for 40 min) took place.

- AF46D (negative control) sample: 1.3 mL NB3 broth mixed with 11.5 g of double pasteurized AF1 sample and incubated for 11 days at 33 °C.
- AF42D sample was fermented as follows: 11.5 g of double pasteurized AF1 sample inoculated with 10^8^ CFUs of *A. kunkeei* strain NADBB37 was re-suspended in 1.3 mL NB3 broth. The sample was mixed and incubated for 11 days at 33 °C.
- AF43D sample was fermented as follows: 11.5 g of double pasteurized AF1 sample inoculated with 10^8^ CFUs of *A. kunkeei* strain NADBB7 was re-suspended in 1.3 mL NB3 broth. The sample was mixed and incubated for 11 days at 33 °C.
- AF44D sample was fermented as follows: 11.5 g of double pasteurized AF1 sample inoculated with 10^8^ CFUs of *A. kunkeei* strain NADBB37, 10^8^ CFUs of *A. kunkeei* strain NADBB7, 10^8^ CFUs *Fructobacillus fructosus* strain NADBB39 and 10^8^ CFUs *Zygosaccharomyces siamensis* was resuspended in 1.3 mL NB3 broth. The sample was mixed and incubated for 11 days at 33 °C.
- AF5U sample was fermented as follows: 11.5 g of AF1 sample inoculated with 10^8^ CFUs of *A. kunkeei* strain NADBB37 was re-suspended in 2.3 mL NB3 broth. The sample was mixed and incubated for 29 days at 33 °C.

### 4.7. Phenotypic Detection of Enzymes Produced by BB Isolates 

In order to test whether BB isolates produce enzymes such as amylases, proteinases, hemicellulases cellulases and enzymes that degrade dyes such as copper oxidases (laccases), manganese oxidases and lignin oxidases [59], PCA plates containing appropriate substrates (see below) were used for qualitative screening. Bacteria were incubated for 1–7 days at 37 °C. Every day, the plates were checked for phenotype detection.

- Amylase activity: PCA + 1% starch. A clear halo was detected around the bacterial colony due to starch breakdown. To confirm amylase activity, the plate was covered with 1% iodine solution for 5–10 min, then washed with deionized water and the presence of light zones around the colony was observed under a light source [60].
- Protease activity: PCA + 2% skimmed milk powder. A clear halo was formed around the bacterial colony due to the breakdown of milk proteins [22].
- Hemicellulose activity (endo-1,4-b-Xylanase): PCA + 0.02% Megazyme AZCL-XYLAN (Megazyme, Bray, Ireland). A blue halo was detected around the bacterial colony due to the breakdown of the hemicellulose granules and the release of the bounded azo pigment [61].
- Cellulose activity (endo-Cellulase): PCA + 0.02% *w*/*v* Megazyme AZCL-HE-CELLULOSE (Megazyme, Bray, Ireland). In the case of cellulase secretion, a blue halo formed around the bacterial colony due to cellulose granule breakdown and the release of the azo pigment [62].
- Guaiacol assay and dye breakdown: PCA + 0.02% *w*/*v* Coomassie Brilliant Blue G (BIO-RAD, Richmond, CA, USA), PCA + 0.02% Coomassie Brilliant Blue R, PCA + 10% Malachite Green (oxalate solution 0.2% *w*/*v*, MERCK, Darmstadt, Germany), PCA + 0.5 mM guaiacol (Alfa Aecar, Kandel, Germany). The secretion of enzymes that break down dyes was detected as a clear halo formed around the bacterial colony. The appearance of brown color in the guaiacol assay indicated the positive phenotype [63].
- In order to determine whether cellulases and hemicellulases are produced and present in mature BB, the assays were carried out using the respective substrates mentioned above for the specific enzymes. Briefly, 6 mm diameter wells were opened in the plates and 100 μL of each protein fraction suspension of BB sample was placed in each of them.

### 4.8. Preparation of BB Protein Fractions

Protein fractions were prepared as follows: 500 mg of each BB sample was dissolved in sterile deionized water (dH_2_O) to a final volume of 1.5 mL. Samples were incubated for 1 h at room temperature with occasional stirring and then centrifuged for 10 min at 10,000× *g*. The supernatant aqueous fraction was collected and solid ammonium sulfate (NH_4_)_2_SO_4_ (AnalaR, BDH Chemicals Ltd., Poole, England) was then added stepwise to a final concentration of 60% *w*/*v*. Samples were mixed gently and left for 20 min at room temperature to equilibrate, then placed for 10 min at −20 °C and centrifuged for 30 min at 11,000× *g*. The supernatant was collected and used in the agar diffusion assays. The pellet was resuspended in 1 mL of sterile dH_2_O.

### 4.9. Detection of Genes Encoding Enzymes in BB Isolates

Biosynthetic genes encoding laccases and hemicellulases produced by BB isolates were partially amplified via PCR. For the laccase gene, the primers Cu1AF and Cu2R [64,65] were used to amplify a 142 bp fragment. PCR reaction mix (50 μL total volume) comprised of 1 mM dNTPs (Invitrogen, Paisley, UK), 1 × Buffer B (Fastgene NIPPON Genetics, Tokyo, Japan), 0.5 mM MgCl_2_ (Fastgene NIPPON Genetics, Tokyo, Japan), 25 pmol of each primer, 3 μL DNA template and 1 unit Taq Polymerase (Fastgene NIPPON Genetics, Tokyo, Japan). The thermal cycler Primus 25 (PEQLAB biotechnologie, Erlangen, Germany) conditions were as follows: 3 min at 95 °C for initial double-strand denaturation, followed by 30 cycles of DNA denaturation at 95 °C for 30 s, primer annealing at 50 °C for 30 s and elongation at 72 °C for 30 s, with a final 5 min extension at 72 °C. The PCR products were checked on a 1.5% (*w*/*v*) agarose gel, stained with ethidium bromide and the bands were visualized under UV light. 

For endoxylanase genes, the degenerated primers GH11R and GH11F [66] were used to amplify a 336 bp fragment. Briefly, the DNA fragment was amplified in a 50 μL reaction mix, comprising 1 μM dNTPs (Invitrogen, Paisley, UK), 1 × Buffer A (Fastgene NIPPON Genetics, Tokyo, Japan), 0.5 mM MgCl_2_ (Fastgene NIPPON Genetics, Tokyo, Japan), 25 pmol of each primer, 3 μL template and 1.5 unit Taq Polymerase (Fastgene NIPPON Genetics, Tokyo, Japan). The thermal cycler Primus 25 (PEQLAB biotechnologie, Erlangen, Germany) conditions for touch-down PCR were as follows: 3 min at 95 °C for initial double-strand denaturation, followed by 30 cycles of DNA denaturation at 95 °C for 30 s, primer annealing at 66 °C–0.5 °C/cyc for 30 s, followed by 25 cycles of DNA denaturation at 95 °C for 30 s, primer annealing at 50 °C for 30 s and elongation at 72 °C for 30 s, with a final 10 min extension at 72 °C. The PCR products were checked on a 1.5% (*w*/*v*) agarose gel, stained with ethidium bromide and the bands were visualized under UV light. 

Amplicons of BB isolates exerting enzyme activity were purified using the NucleoSpin Gel and PCR clean-up kit (Macherey-Nagel, Düren, Germany) and directly sequenced via Sanger dideoxy termination method by Cemia (Larissa, Greece). Chromas version 2.6.6 software (Technelysium Pty Ltd., South Brisbane, Australia, www.technelysium.com.au (last accessed 5 June 2024)) was used to check the quality of the obtained sequencing data. Sequences were processed using Gene Runner version 6.5 software (www.generunner.net (last accessed 5 June 2024)) and subjected to a BlastN (Megablast) analysis (https://blast.ncbi.nlm.nih.gov/Blast.cgi (last accessed 5 June 2024)) to identify sequences with the highest similarity.

### 4.10. Determination of Minimum Inhibitory Concentration (MIC) and Minimum Bactericidal Concentration (MBC)

The determination of minimum inhibitory concentration (MIC) was carried out in sterile 96-well polystyrene microtiter plates (Kisker Biotech GmbH & Co. KG, Steinfurt, Germany) using a spectrophotometric bioassay as described by Didaras et al., 2021. Briefly, 0.5 g of BCP and artificially fermented BCP samples were suspended in sterile ddH_2_O (2 mL final volume) for one hour at room temperature with occasional vortex and then centrifuged at 10,000× *g* for 7 min. The aqueous phase was filtered through a 0.22 μM filter and used for serial dilutions in Mueller–Hinton broth corresponding from 25 to 0.39% *v*/*v*. Overnight bacterial cultures grown in Mueller–Hinton broth were adjusted to a 0.5 McFarland turbidity standard (~1.5 × 10^8^ CFU/mL). Ten μL Mueller–Hinton broth containing approximately 5 × 10^4^ CFUs were added to 190 μL of tested 2-fold sample dilutions.

Mueller–Hinton broth inoculated only with pathogens in the absence of BCP serial dilutions was used as a positive control. Wells containing only Mueller–Hinton broth, not inoculated with bacteria, were monitored to test for contamination.

The optical density (OD) was determined at 600 nm using a PerkinElmer Multimode plate reader, (PerkinElmer, Inc., 940 Winter Street, Waltham, MA, USA) just prior to incubation (t = 0) and 24 h after incubation (t = 24) at 37 °C. The OD for each negative control replicate well at t = 24 was subtracted from the OD of the same replicate test well with bacteria at t = 24. The growth inhibition at each BCP dilution was determined using the formula % inhibition = [1 − (OD test well—OD of corresponding negative BB control well)] × 100. MIC was determined as the lowest bee bread concentration which results in 100% growth inhibition. 

The MBC was determined by transferring a small quantity of sample contained in each replicate well of the microtiter plates to Mueller–Hinton agar plates by using a microplate replicator (BoekelScientific, Feasterville-Trevose, PA, USA). The plates were incubated at 37 °C for 24 h. The MBC was determined as the lowest concentration at which no colonies were observed [16].

### 4.11. Identification of Culturable Bacteria and Yeasts via 16S rRNA Gene and ITS SANGER Sequencing, Respectively 

The identification of bacteria via 16S rRNA gene sequencing was performed as previously described [23]. Briefly, bacterial genomic DNA was extracted using the ExtractMe Genomic DNA Kit (Blirt, Gdansk, Poland). The 16S rRNA gene was amplified through PCR using the universal primers 27F (5′-AGAGTTTGATCMTGGCTCAG-3′) and 1492R (5′-GGTTACCTTGTTACGACTT-3′) (Eurofins Genomics, Ebersberg, Germany). PCR conditions: initial denaturation at 95 °C for 3 min, followed by 35 cycles of denaturation at 95 °C for 30 s, annealing at 50 °C for 30 s and elongation at 72 °C for 2 min. A final elongation step at 72 °C for 2 min was added (thermal cycler Primus 25, PEQLAB biotechnologie, Erlangen, Germany). For ITS amplification ITS1 (5′-TCCGTAGGTGAACCTGCGG-3′) and ITS4 (5′-TCCTCCGCTTATTGATATGC-3′) (Eurofins Genomics, Ebersberg, Germany) were used. The reaction mixture and PCR conditions were performed as described previously [23]. Amplicons were purified using the NucleoSpin Gel and PCR clean-up kit (Macherey-Nagel, Düren, Germany) and sequenced via the Sanger dideoxy termination method by Macrogen Europe (Amsterdam, The Netherlands). Yeast amplicons were sequenced via the Sanger dideoxy termination method by CeMIA SA (Larissa, Greece). Chromas version 2.6.6 software (Technelysium Pty Ltd., South Brisbane, Australia, www.technelysium.com.au (last accessed 5 June 2024)) was used to check the quality of the obtained sequencing data. Sequences were assembled into a single sequence via MEGA X version 10.1.6 software (Kumar et al., 2018 [67]) and Gene Runner version 6.5 software (www.generunner.net, accessed on 21 June 2022) and subjected to BlastN (Megablast) (https://blast.ncbi.nlm.nih.gov/Blast.cgi (last accessed 5 June 2024)) search in the 16S rRNA Database-GENEBANK to identify sequences with the highest similarity. Obtained sequences were deposited to GenBank under the following accession numbers ON911235-ON911282.

## 5. Conclusions

Most consumers are not familiar with bee bread, a honey bee product that should be considered a functional food due to its high content of nutrients (essential amino acids, sugars, fatty acids including ω-3, polyphenols, vitamins, macro and microelements) and exerted bioactivity (antioxidant, antimicrobial, anti-inflammatory). This study aims to investigate the microbiota during bee bread maturation and to elucidate the role of autochthonous microorganisms in the conversion of BCP to BB as well as exerted bioactivity. Our data demonstrated that during BB maturation microbial diversity dynamically fluctuated probably due to microbial competition and adaptation to highly volatile conditions, ending up rather stable in terms of relative abundance and microbial community structure. Implementing culture-dependent methods demonstrated that bacteria isolated from BB produce and secrete numerous enzymes, thus indicating a high biosynthetic potential. It is very plausible that the production of lignin-degrading enzymes (laccases or oxidases) and cellulases by BB bacteria breaches pollen grain walls. In that way, nutrients and bioactive compounds contained in pollen grains are further released, thus contributing to higher bioactivity exerted by BB compared to fresh BCP.

In this study, characterized bacteria isolated from BB have demonstrated antibacterial activity against human pathogens, thus indicating BB microbiota contribution to exerted bioactivity. Furthermore, it has been shown the antibacterial activity of *Apilactobacillus* and *Fructobacillus* strains isolated from BB for the first time. Secondary metabolites such as non-ribosomal peptides, polyketides and other antimicrobial compounds produced by BB microbiota during BCP fermentation and BB maturation could directly contribute to antimicrobial activity exerted by BB. This is further supported by our findings that artificial fermentation of BCP by indigenous microbial strains increased the MIC and MBC values against tested pathogens. 

Overall, further studies on BB microorganisms regarding the antimicrobial activity as well as the biosynthetic and probiotic potential could lead to novel biotechnological applications in medicine (discovery of novel antimicrobial agents), in agriculture (production of novel functional food supplements for animals, or implementation of BB microbes for converting agriculture byproducts to added value products) as well as in human nutrition to discovery of novel enzymes implemented in paper industry for example. Furthermore, research may lead to a variety of products depending on BCP botanical origin and implemented microbial stains as a starter culture in artificial fermentation at an industrial scale.

## Figures and Tables

**Table 1:** Antibacterial activity exerted by *Apilatobacillus* and *Fructobacillus* strains.

Accession Number (GenBank)	*K. pneumoniae* (cm ± ^1^SD)	*S. enterica* ser. Typhimurium (cm ± ^1^SD)	*P. aeruginosa* (cm ± ^1^SD)	*S. aureus* (cm ± ^1^SD)	% Identity	Identified Bacteria	16 S rRNA Gene Sequence (bp)	Strain
ON911239	3 ± 0.7	2.7 ± 0.9	3 ± 0.8	0.8 ± 0.1	99.79	*Apilactobacillus kunkeei*	1446	NADBB5
ON911241	3.1 ± 0.7	2.7 ± 0.9	3 ± 0.8	0.6 ± 0.1	100.00	*Apilactobacillus kunkeei*	1452	NADBB7
ON911243	3.2 ± 0.2	2.7 ± 0.4	3 ± 0.1	0	99.86	*Apilactobacillus kunkeei*	1424	NADBB9
ON911252	3.5 ± 0.5	3.4 ± 0.7	2.4 ± 1.4	0.7 ± 0.1	100.00	*Apilactobacillus kunkeei*	1447	NADBB20
ON911258	2.8 ± 1	3.4 ± 0.4	2.7 ± 1.2	0.9 ± 0	99.79	*Apilactobacillus kunkeei*	1412	NADBB27
ON911261	3 ± 0.9	2.8 ± 1	3 ± 0.8	0.7 ± 0.3	99.86	*Apilactobacillus kunkeei*	1433	NADBB30
ON911265	3 ± 1.3	3.3 ± 0.8	2.9 ± 1	0.9 ± 0.2	100.00	*Apilactobacillus kunkeei*	1413	NADBB34
ON911266	3.4 ± 0.8	3.2 ± 1.2	3.3 ± 0.8	1.6 ± 0.4	100.00	*Apilactobacillus kunkeei*	1208	NADBB35
ON911268	3.3 ± 0.9	3 ± 1.3	3.4 ± 0.9	1.1 ± 0.2	100.00	*Apilactobacillus kunkeei*	1446	NADBB37
ON911269	3.3 ± 0.9	3.1 ± 1.2	3.3 ± 1	1.1 ± 0.1	100.00	*Apilactobacillus kunkeei*	1443	NADBB38
ON911270	2.7 ± 0.6	1.8 ± 0.3	2.4 ± 0.4	0	99.43	*Fructobacillus fructosus*	1393	NADBB39
ON911279	3.3 ± 0.9	3 ± 1.2	3.3 ± 1	1.1 ± 0.4	100.00	*Apilactobacillus kunkeei*	1447	NADBB48

All tests have been performed at least in triplicates, ^1^SD: standard deviation.

**Table 2:** Antibacterial activity and biosynthetic potential of bacteria isolated from BB.

Accession Number (GenBank)	Biosynthetic Potential	Antibacterial Activity	% Identity	Identified Bacteria	16S rRNA Gene Sequence (bp)	Strain
ON911235	A, P, R, G, **L**	1 *, 3 *	100.00	*Pseudomonas αeruginosa*	1400	NADBB1
ON911236	X, **X**, A, P, G, M	1 ***, 3 ***, 4 ***	100.00	*Bacillus* sp.	1395	NADBB2
ON911237	A, P, G, M, **L**	1 *, 3 *, 4 ***	99.93	*Bacillus thuringiensis*	1400	NADBB3
ON911238	A, P, R, G, **L**	1 ***, 2 ***, 3 ***	100.00	*Bacillus cereus*	1404	NADBB4
ON911240	C, X, A, P, GUA	1 **, 3 *, 4 *	100.00	*Bacillus siamensis*	1426	NADBB6
ON911242	X, A, G, M	1 **, 4 ***	100.00	*Bacillus* sp.	1416	NADBB8
ON911244	A, G, **L**	3 **, 4 *	99.86	*Bacillus thuringiensis*	1389	NADBB10
ON911245	X, **X**, **X**, A, P, G, M,	1 ***, 4 ***	99.93	*Bacillus* sp.	1417	NADBB11
ON911246	A, P, R, G, **L**	3 ***	99.93	*Bacillus thuringiensis*	1390	NADBB13
ON911247	C, X, **X**, P, R	1 **, 4 *	99.93	*Bacillus* sp.	1376	NADBB14
ON911248	C, A, P, G, M	1 **, 3 *, 4 ***	99.57	*Bacillus licheniformis*	1400	NADBB16
ON911249	C, X, A, P, M, **L**	1 **	100.00	*Bacillus subtilis*	1425	NADBB17
ON911250	A, P, G, M, **L**	1 **, 3 *, 4 *	99.86	*Bacillus* sp.	1419	NADBB18
ON911251	C, X, A, G, **L**, **L**		100.00	*Bacillus subtilis*	1423	NADBB19
ON911253	A, P, R, G, **L**, L	3 **	99.93	*Bacillus cereus*	1398	NADBB21
ON911254	X, **X**, P, G, M,	1 **	100.00	*Bacillus* sp.	1420	NADBB23
ON911255	C, X, **X**, **X**, P, G	1 **, 3 ***, 4 ***	100.00	*Bacillus* sp.	1414	NADBB24
ON911256	C, X, A, P, M	1 *	100.00	*Bacillus siamensis*	1410	NADBB25
ON911257	C, X, A, P, G, M	1 *, 3 *, 4 *	100.00	*Bacillus subtilis*	1392	NADBB26
ON911259	A, G, M, **L**		99.93	*Bacillus* sp.	1418	NADBB28
ON911260	A, P, G	1 **, 2 ***, 3 ***, 4 ***	100.00	*Bacillus cereus*	1402	NADBB29
ON911262	A, M, **L**		99.25	*Staphylococcus hominis*	1341	NADBB31
ON911263	C, X, **X**, P, G, M	1 ***, 3 ***, 4 ***	100.00	*Bacillus* sp.	1371	NADBB32
ON911264	C, X, A, P, M, G, **L**	1 ***, 3 ***	99.86	*Bacillus siamensis*	1417	NADBB33
ON911267	A, P, G, **L**, **L**	1 *, 3 *, 4 *	100.00	*Bacillus thuringiensis*	1313	NADBB36
ON911271	C, X, **X**, P, R, G, **L**	1 ***, 3 **, 4 ***	100.00	*Bacillus safensis*	1416	NADBB40
ON911272	C, X, **X**, P, G	1 ***, 4 ***	99.93	*Bacillus* sp.	1419	NADBB41
ON911273	C, X, A, P, G	1 **, 3 ***, 4 ***	99.71	*Bacillus halotolerans*	1393	NADBB42
ON911274	C, A, P, G, M	1 *, 4 **	99.86	*Bacillus licheniformis*	1395	NADBB43
ON911275	X, P, R, G	1 **, 4 **	100.00	*Bacillus safensis*	1383	NADBB44
ON911276	C, X, **X**, A, M;	1 *	100.00	*Bacillus pumilus*	1391	NADBB45
ON911277	C, X, P, R, G, M	1 **, 3 ***	100.00	*Bacillus aerophilus*	1412	NADBB46
ON911278	A, P, R, G, **L**	3 ***	100.00	*Bacillus thuringiensis*	1396	NADBB47
ON911280	X, **X**, **X**, P, G	1 *	99.85	*Bacillus safensis*	1323	NADBB49
ON911281	X, **X**, **X**, P, G	1 **, 3 **, 4 **	99.93	*Bacillus safensis*	1379	NADBB50

**Antimicrobial activity against** 1: *S aureus*, 2: *P. aeruginosa*, 3: *S. enterica* ser. Typhimurium, 4: *K. pneumoniae*. *: 3–7 mm, **: 7–11 mm, ***: >11 mm. **Biosynthetic potential:** C: cellulase positive phenotype, X: xylanase positive phenotype, **X**: endoxylanase gene confirmed via sequencing (336 bp fragment), **X**: endoxylanase gene PCR detection, A: amylase positive phenotype, P: proteinase positive phenotype, R: coomassie brilliant blue red degradation, G: coomassie brilliant blue green degradation, M: malachite green degradation, **L**: laccase gene confirmed via sequencing (142 bp fragment), **L**: laccase gene PCR detection.

**Table 3:** MIC and MBC values of double pasteurized and artificially fermented BCP.

*S. enterica* ser. Typhimurium		*S. aureus*		Samples
MBC *w*/*v*	MIC *w*/*v*	MBC *w*/*v*	MIC *w*/*v*	
>12.5%	12.5%	>12.5%	12.5%	AF1
>12.5%	>12.5%	>12.5%	12.5%	AF46D
>12.5%	6.25%	>12.5%	12.5%	AF42D
12.5%	6.25%	>12.5%	12.5%	AF43D
>12.5%	12.5%	>12.5%	12.5%	AF44D
6.25%	6.25%	12.5%	6.25%	AF5

**Table 4:** Botanical origin, location and harvest month of BCP and BB samples.

Botanic Origin	Region	Month	Age of BB and BCP Samples	Samples
Multifloral	Pelion, Thessaly	May	1-day-old BCP	S1 and S6
Multifloral	Pelion, Thessaly	May	1-day-old BB	S2 and S7
Multifloral	Pelion, Thessaly	May	11-day-old BB	S3 and S8
Multifloral	Pelion, Thessaly	June	27-day-old BB	S4 and S9
Multifloral	Pelion, Thessaly	June	41-day-old BB	S5
Multifloral	Rethymno, Crete	May	Not determined	S11
Multifloral	Thessaly	May	BCP 24 h	AF1

## Data Availability

All data regarding this study are available in the article and in its online Supplementary Materials.

## Supplementary Materials

The following supporting information can be downloaded at https://www.mdpi.com/article/10.3390/ph17060761/s1, Figure S1: Rarefaction curve of 16S data for samples S1–S5; Figure S2: Rarefaction curve of ITS data for samples S6–S9; Table S1: Basic statistics of each sample; Table S2: Alpha diversity metrics for bacteria; Table S3: Alpha diversity metrics for fungi; Table S4: Relative abundance of bacterial families/genera in samples S1–S5; Table S5: Relative abundance of fungal families/genera in samples S6–S9; Table S6: Bray-Curtis beta-diversity matrix for bacteria (samples S1–S5); Table S7: Jaccard beta-diversity matrix for bacteria (samples S1–S5); Table S8: Weighted Unifrac beta-diversity matrix for bacteria (samples S1–S5); Table S9: Unweighted Unifrac beta-diversity matrix for bacteria (samples S1–S5); Table S10: Bray-Curtis beta-diversity matrix for fungi (samples S6–S9); Table S11: Jaccard beta-diversity matrix for fungi (samples S6–S9); Table S12: Weighted Unifrac beta-diversity matrix for fungi (samples S6–S9); Table S13: Unweighted Unifrac beta-diversity matrix for fungi (samples S6–S9); Table S14: Enzyme fragment amplicons sequences.
