# Supplementary material for: Contribution of Microbiota to Bioactivity Exerted by Bee Bread
Source: Pharmaceuticals (Basel). 2024 Jun 11;17(6):761. doi: 10.3390/ph17060761 (PMC11206572; doi:10.3390/ph17060761)
Supplement: Supplementary file 1 [file pharmaceuticals-17-00761-s001.zip › pharmaceuticals-2973587-supplementary.pdf]

Supplementary Table S1. Basic statistics of each sample

Sample	S1	S2	S3	S4	S5	S6	S7	S8	S9
Sample de	Pollen	Beebread	Beebread	Beebread	Beebread	Pollen	Beebread	Beebread	Beebread
Sequence	16S	16S	16S	16S	16S	ITS	ITS	ITS	ITS
Total reads	249440	236088	267792	269312	210454	373528	356546	389092	393676
GC(%)	55.58	55.4	54.99	55.02	55.03	49.23	52.4	49.89	47.13
AT(%)	44.42	44.6	45.01	44.98	44.97	50.77	47.6	50.11	52.87
Q20(%)	93.8	94.07	94.25	94.66	93.59	94.99	93.98	92.04	91.82
Q30(%)	86.12	86.49	86.89	87.47	85.74	88.61	86.7	84.85	84.21
Read pairs	124720	118044	133896	134656	105227	186764	178273	194546	196838
Reads pai	122018	115435	130782	132207	102125	75891	134286	137775	130956
f ltered	72602	70506	81815	83099	60677	42365	79456	85530	81686
denoised	72379	70299	80873	82265	60334	42269	79153	85284	81470
merged	71788	69110	77463	78867	59154	40914	74882	80938	74888
non-chim	10537	10871	11391	9048	7353	40486	73303	79837	74056

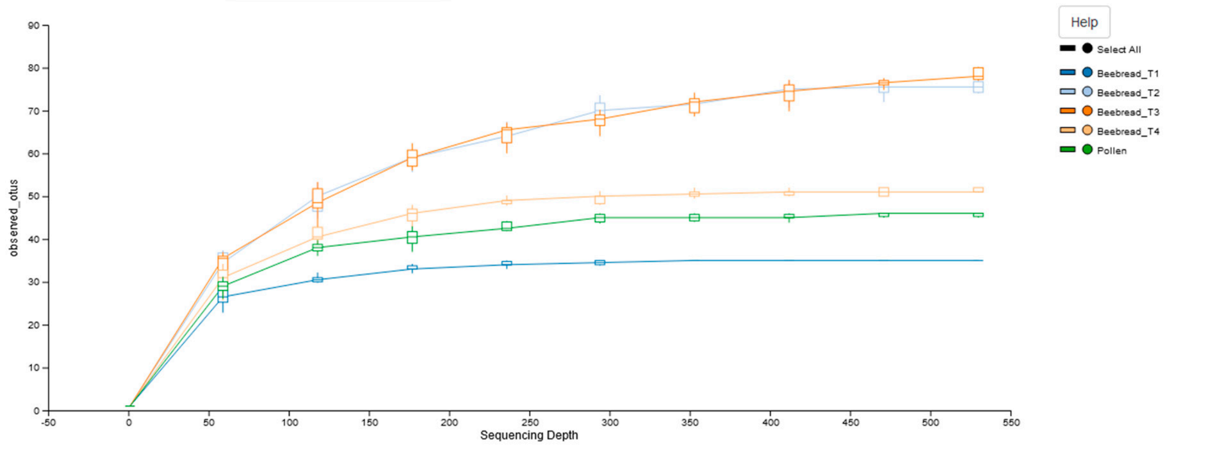

Supplementary Figure S1. Rarefaction curve of 16S data for samples S1-S5.

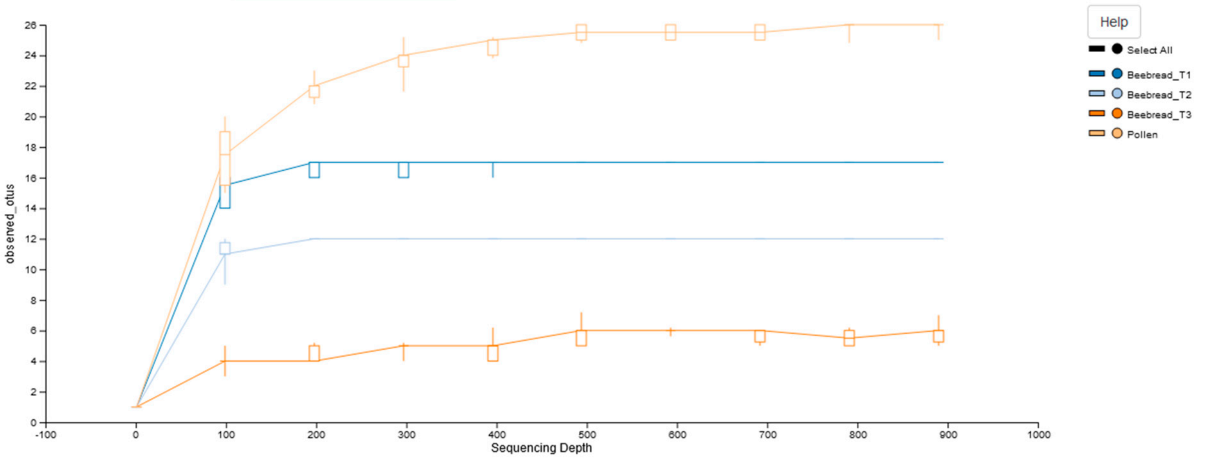

Supplementary Figure S2. Rarefaction curve of ITS data for samples S6-S9.

Supplementary Table S2. Alpha diversity metrics for bacteria.

sample-id	shannon	observed_	faith_pd	pielou_ev	chao1_ci
S1	5.121	46	4.864	0.927	(46, 46.00
S2	4.778	35	4.239	0.931	(35, 35.00
S3	5.714	76	7.271	0.914	(80, 80.00
S4	5.603	80	7.693	0.886	(82, 82.00
S5	5.146	52	6.012	0.903	(52, 52.00

Supplementary Table S3. Alpha diversity metrics for fungi.

sample-id	shannon	observed_	faith_pd	pielou_ev	chao1_ci
S6	3.380	26	6.531	0.719	(26, 26.0)
S7	3.367	17	4.612	0.824	(17, 17.00
S8	2.843	12	3.455	0.793	(12, 12.0)
S9	0.586	6	1.497	0.227	(8, 8.0)

Supplementary Table S4. Relative abundance of bacterial families/genera in samples S1-S5.

Phylum	Bacterial families/genera	S1 %	S2 %	S3 %	S4 %	S5 %
Actinobacteria	Corynebacteriaceae;Corynebacterium 1	0.000	0.000	0.000	3.222	1.330
Bacteroidetes	Hymenobacteraceae;Hymenobacter	9.711	5.400	2.717	1.134	0.000
Bacteroidetes	Sphingobacteriaceae;Mucilaginibacter	6.474	2.421	0.242	0.537	1.284
Bacteroidetes	Muribaculaceae;uncultured bacterium	0.000	0.000	0.000	3.162	1.696
Bacteroidetes	Dysgonomonadaceae;Dysgonomonas	0.000	0.000	0.000	0.418	3.072
Firmicutes	Lactobacillaceae;Lactobacillus	4.277	7.635	6.703	1.134	4.952
Proteobacteria	Sphingomonadaceae;Sphingomonas	6.243	11.359	8.152	6.086	13.343
Proteobacteria	Pseudomonadaceae;Pseudomonas	4.624	1.117	6.099	14.141	4.677
Proteobacteria	Burkholderiaceae;Massilia	13.064	2.980	5.857	4.356	2.934
Proteobacteria	Moraxellaceae;Acinetobacter	6.127	7.263	5.193	3.162	6.419
Proteobacteria	Enterobacteriaceae;Unknown genus	9.249	3.352	1.691	3.103	5.365
Proteobacteria	Enterobacteriaceae;Pantoea	0.000	6.890	7.367	3.043	1.100
Proteobacteria	Acetobacteraceae;Unknown genus	0.000	0.000	7.548	7.936	1.880
Proteobacteria	Enterobacteriaceae;Phaseolibacter	0.809	4.842	1.510	7.041	1.605
Proteobacteria	Enterobacteriaceae;Enterobacter	4.624	7.076	0.000	0.000	0.000
Proteobacteria	Beijerinckiaceae;1174-901-12	0.000	6.704	3.442	0.000	1.055
Proteobacteria	Orbaceae;Gilliamella	0.000	0.000	3.502	3.162	2.889
Proteobacteria	Burkholderiaceae;Unknown genus	0.000	0.559	2.053	0.716	4.264
Proteobacteria	Rhodanobacteraceae;Luteibacter	4.046	2.607	0.000	0.000	0.000
Proteobacteria	Orbaceae;Ambiguous_taxa	0.000	1.490	0.000	3.401	0.779
Proteobacteria	Enterobacteriaceae;Rosenbergiella	0.000	0.000	3.019	1.611	0.000
Tenericutes	Entomoplasmataceae;Mesoplasma	4.277	7.263	2.234	3.282	0.000
	Other	26.474	21.043	32.669	29.356	41.357

Supplementary Table S5. Relative abundance of fungal families/genera in samples S6-S9.

Phylum	Fungi families / genera	S6 %	S7 %	S8 %	S9 %
Ascomycota	Saccharomycetales;Saccharomycetaceae;Zygosaccharomyces	51.639	10.275	67.929	99.581
Ascomycota	Hypocreales;Hypocreaceae;Trichoderma	1.004	36.227	5.568	0.126
Ascomycota	Saccharomycetales; Unknown sp. sp.	2.930	17.095	5.568	0.207
Ascomycota	Pleosporales;Pleosporaceae;Alternaria	8.320	5.669	5.122	0.000
Ascomycota	Glomerellales;Glomerellaceae;Unknown sp. sp.	0.000	4.960	0.000	0.000
Ascomycota	Chaetothyriales;Herpotrichiellaceae;Unknown sp.	0.000	4.606	0.000	0.000
Ascomycota	Pleosporales;Phaeosphaeriaceae;Leptospora	0.000	0.000	3.898	0.000
Ascomycota	Rhyt ismatales;Rhyt ismataceae;Colpoma	0.000	0.000	3.007	0.000
Basidiomycota	Agaricales;Physalacriaceae;Cryptomarasmius	0.000	7.529	1.782	0.000
Basidiomycota	Hymenochaetales;Hymenochaetaceae;Fuscoporia	3.156	3.189	0.000	0.000
Basidiomycota	Agaricales;Lycoperdaceae;Lycoperdon	0.000	4.074	0.000	0.000
Basidiomycota	Agaricales; Unknown sp. sp.	3.852	0.000	0.000	0.000
Basidiomycota	Cystof lobasidiales;Mrakiaceae;Krasilnikovozyma	3.832	0.000	0.000	0.000
Basidiomycota	Agaricales;Psathyrellaceae;Unknown sp.	3.340	0.000	0.000	0.000
Basidiomycota	Unknown sp.	3.176	0.000	0.000	0.000
Basidiomycota	Malasseziales;Malasseziaceae;Malassezia	3.012	0.000	0.000	0.000
Basidiomycota	Cantharellales;Cantharellaceae;Craterellus	0.000	0.000	3.007	0.000
	Other	15.738	6.377	4.120	0.086

Supplementary Table S6. Bray-Curtis beta-diversity matrix for bacteria (samples S1-S5).

	S1	S2	S3	S4	S5
S1	0.000	0.658	0.740	0.791	0.789
S2	0.658	0.000	0.715	0.821	0.830
S3	0.740	0.715	0.000	0.638	0.681
S4	0.791	0.821	0.638	0.000	0.745
S5	0.789	0.830	0.681	0.745	0.000

Supplementary Table S7. Jaccard beta-diversity matrix for bacteria (samples S1-S5).

	S1	S2	S3	S4	S5
S1	0.000	0.791	0.870	0.865	0.911
S2	0.791	0.000	0.844	0.883	0.885
S3	0.870	0.844	0.000	0.836	0.847
S4	0.865	0.883	0.836	0.000	0.832
S5	0.911	0.885	0.847	0.832	0.000

Supplementary Table S8. Weighted Unifrac beta-diversity matrix for bacteria (samples S1-S5).

	S1	S2	S3	S4	S5
S1	0.000	0.172	0.196	0.209	0.207
S2	0.172	0.000	0.164	0.199	0.193
S3	0.196	0.164	0.000	0.167	0.173
S4	0.209	0.199	0.167	0.000	0.182
S5	0.207	0.193	0.173	0.182	0.000

Supplementary Table S9. Unweighted Unifrac beta-diversity matrix for bacteria (samples S1-S5).

	S1	S2	S3	S4	S5
S1	0.000	0.601	0.650	0.664	0.661
S2	0.601	0.000	0.575	0.649	0.661
S3	0.650	0.575	0.000	0.644	0.612
S4	0.664	0.649	0.644	0.000	0.639
S5	0.661	0.661	0.612	0.639	0.000

Supplementary Table S10. Bray-Curtis beta-diversity matrix for fungi (samples S6-S9).

	S6	S7	S8	S9
S6	0.000	0.772	0.416	0.961
S7	0.772	0.000	0.718	0.942
S8	0.416	0.718	0.000	0.908
S9	0.961	0.942	0.908	0.000

Supplementary Table S11. Jaccard beta-diversity matrix for fungi (samples S6-S9).

	S6	S7	S8	S9
S6	0.000	0.735	0.813	0.815
S7	0.735	0.000	0.682	0.789
S8	0.813	0.682	0.000	0.714
S9	0.815	0.789	0.714	0.000

Supplementary Table S12. Weighted Unifrac beta-diversity matrix for fungi (samples S6-S9).

	S6	S7	S8	S9
S6	0.000	0.561	0.251	0.523
S7	0.561	0.000	0.612	0.768
S8	0.251	0.612	0.000	0.401
S9	0.523	0.768	0.401	0.000

Supplementary Table S13. Unweighted Unifrac beta-diversity matrix for fungi (samples S6-S9).

	S6	S7	S8	S9
S6	0.000	0.485	0.662	0.780
S7	0.485	0.000	0.563	0.715
S8	0.662	0.563	0.000	0.661
S9	0.780	0.715	0.661	0.000

Supplementary Table S14. Enzyme fragment amplicons sequences

**Accession number (GenBank)**	**Strain**	**Enzyme**	**Enzymes fragment PCR amplicons sequences**
ON911253 (B. cereus)	NADBB21	Laccase	TAATGGATTCCAGGTGTGACGCAA GATGCGGTTGACCTGGAAAAAGTT TCACCTATGAATTTGAAGCGAACGT ACCAGGMACYTTCTGGTACCACAG
ON911267 (B. thuringiensis)	NADBB36	Laccase	GCGTGACACAAGATGCAGTTGAA CCAGGAAAAAGTTTCACTTACGAA TTTGAAGCGAACGTACCAGGMACY TTCTGGTACCACAGC
ON911251 (*B. subtilis)*	NADBB19	Laccase	GCCCATGCTCACGCAGTGCCCCAGC GCGGCGGACTCCTCGTTCCTGTACA ACTTCACCGTCGACCAGCCGGGMA CYTTCKGGWACCA
ON911245 (*Bacillus* sp.*)*	NADBB11	Endoxylanase	GGGGCACATATCGTCCAACAGGAG CGTATAAAGGATCATTCTATGCAG ATGGAGGCACATATGACATTTATG AAACGACCCGCGTTAATCAGCCTT CCATTATCGGGATCGCGACCTTCA AGCAATATTGGAGTGTACGACAA

			ACAAAACGTACAAGCGGAACGG TCTCCGTCAGTGCGCATTTTAATA AATGGGAAAGCTTAGGCATGCCA ATGGGTAAAATGTATGAAACAGC ATTTACTGTAGARGGSTAYMAAA GSAGSGGCA
ON911255 (*Bacillus* sp.)	NADBB24	Endoxylanase	ACATTGTTGATTCATGGGGCACAT ATCGTCCAACAGGAGCGTATAAA GGATCATTCTATGCAGATGGAGG CACATATGACATTTATGAAACGA CCCGCGKTAATCAGCCTTCCATTA TCGGGATCGCGACCTTCAAGCAAT ATTGGAGTGKACGACAAACAAAA CGTACAAGCGGAACGGTCTCCGTCA GTGSGCATTTTAATAAATGGGAAAG CTTARGCATGCCAATGGGTAAAATG KATGAAACARCATTTACTGKARAAG GSTACCAAA
ON911280 (*B. safensis*)	NADBB49	Endoxylanase	TGATTCATGGGGCACATATCGTCCA ACAGGAGCGTATAAAGGATCATTCT ATGCAGATGGAGGCACATATGACAT TTATGAAACGACCCGCGTTAATCAGC CTTCCATTATCGGGATCGCGACCTTCA AGCAATATTGGAGTGKACGACAAACA AAASAGTACAAGCGGAACGGTCTCCG TCAGTGCGCATTTTAATAAATGGGAAA GCTTAGGCATGCCAATGGGTAAAATGT ATGAAACAGCATTTACTGKAGAAGGST ACCAAAGCAGCGGCA
